# Heart Failure Is Not Associated with a Poor Outcome after Mechanical Thrombectomy in Large Vessel Occlusion of Cerebral Arteries

**DOI:** 10.1155/2019/4695414

**Published:** 2019-08-01

**Authors:** Marlena Schnieder, Anneki von Glasenapp, Amelie Hesse, Marios N. Psychogios, Mathias Bähr, Gerd Hasenfuß, Marco R. Schroeter, Jan Liman

**Affiliations:** ^1^Department of Neurology, University Medical Center Göttingen, 37075 Göttingen, Germany; ^2^Clinic of Cardiology & Pneumology/Heart Center, University Medical Center Göttingen, 37075 Göttingen, Germany; ^3^Department of Neuroradiology, University Medical Center Göttingen, 37075 Göttingen, Germany

## Abstract

The impact of heart failure on outcome in stroke patients is not fully understood. There is evidence for an increased mortality and morbidity, but it remains uncertain whether thrombectomy in patients with large vessel occlusion (LVO) in the anterior circulation is less effective in patients with heart failure compared to patients without. Retrospectively, we analyzed echocardiographic data of all patients in our stroke database, who underwent mechanical thrombectomy (n=668) for the presence of heart failure. Furthermore, we collected baseline characteristics and neurological and neuroradiological parameters. In the analysis, 373 of the 668 patients of our stroke database underwent echocardiography. Of these 373 patients, 90 patients (24%) suffered from heart failure with reduced left ventricular ejection fraction measured by echocardiography according to the current guidelines. After adjustment for age, the Alberta stroke program early CT score (ASPECTS), and time from symptom onset to recanalization, the analysis revealed that thrombectomy in patients with heart failure and LVO is not associated with less favorable outcome measured by the modified Rankin Scale after 90 days (3 (0-6) vs. 3 (1-5); p=0.380). Moreover, we could not find a significant difference in mortality compared to patients without heart failure (11.0% vs. 7.4%; p=0.313).

## 1. Introduction

Heart failure is a disease associated with a high mortality and morbidity [1, 2]. Moreover, most of the patients with symptomatic heart failure suffer from comorbidities, such as peripheral artery embolism, pulmonary embolism, and stroke, leading to a higher mortality [3].

The risk of stroke in heart failure increases with the severity of heart failure, and patients with a moderate heart failure have an annual risk of stroke of approximately 1.5% [4], leading up to a risk of 4% in severe heart failure [5].

Because ischemic stroke and heart failure share common risk factors, such as diabetes and arterial hypertension [6], interactions between the two diseases are likely. Moreover, heart failure leads to a hypercoagulate state with rheological alterations due to decreased blood flow [7] leading to a higher rate of stroke in patients with heart failure compared to the normal population [8]. These patients also have a higher incidence of atrial fibrillation and the risk of atrial fibrillation could vice versa increase the severity of heart failure [9]. It has been shown that, in patients with heart failure suffering from stroke, outcome is worse compared to those without heart failure, displayed by higher mortality, but also reduced neurological functional outcome [10]. Patients with heart failure may have a worse outcome after mechanical thrombectomy, since it could be shown that these patients have a poorer collateral-status regarding the blood supply of the brain compared to the patients without heart failure [11].

In contrast the analysis of the VISTA cohort could show that systemic thrombolysis with intravenous recombinant tissue plasminogen activator (rt-PA) is as effective in patients with heart failure as in those without [12].

Thus, it remains uncertain whether mechanical thrombectomy in patients with large vessel occlusion is less effective in patients with heart failure compared to patients with normal systolic left ventricular heart function.

## 2. Methods

We retrospectively analyzed the echocardiographic data of patients of our prospectively kept stroke database, regarding the presence of heart failure. Patients were classified as suffering from heart failure when showing a reduced ejection fraction < 55% in the echocardiography.

The collected data includes neurological features as the National Institute of Health Stroke Scale (NIHSS) and the modified Rankin Scale (mRS) and neuroradiological data such as the Alberta stroke program early CT score (ASPECTS) and the Collateral Status. The NIHSS and the mRS were assessed by experienced stroke neurologist and the ASPECTS and the collateral stroke by a senior neuroradiologist. The rate of symptomatic intracranial hemorrhage was collected as well. A symptomatic intracranial hemorrhage was defined as a deterioration of ≥ 4 in the NIHSS [13]. Furthermore, we collected the cardiovascular risk factors (CVRF) as well as long-term holter electrocardiographic data and duplex sonography of the brain-supplying arteries. Antiplatelet medication and anticoagulation were collected as well as heart failure medication.

Transthoracic echocardiography was performed by experienced cardiological examiners using a standard operating procedure (IE33, CX50 and X7-2t probe (Philips Medical Systems, Eindhoven, Netherlands) or Vivid E9 and 6VT-D probe (GE Healthcare, USA)). We collected echocardiography data such as the ejection fraction, the diameter of atrial and ventricle, wall motion abnormalities, patent foramen ovale, and valve insufficiencies and stenosis. The valve insufficiencies and stenosis were further graduated into mild, moderate, and severe.

The statistical analysis was performed in IBM SPSS Statistics 24 package (IBM, Armonk, New York, USA). Descriptive analysis was descripted in relative frequencies using mean and median and standard deviation or the interquartile range. Group comparisons were performed using chi-square test. If they are not normally distributed, Mann-Whitney U-Test was performed. Multivariate regression analysis was performed to analyze the impact of the heart failure on the outcome of the patients. Possible baseline characteristics associated with the outcome (p<0.1) were included in the analysis. A backwards selection was then applied to find the best set of predictors. A p-value of < 0.05 was considered statistically significant.

## 3. Results

We screened 668 patients of our prospectively kept stroke database of patients with a large vessel occlusion who underwent mechanical thrombectomy for the presence of heart failure and further echocardiological characteristics. In total 373 (55.8%) patients underwent echocardiography. 90 (24%) of these patients suffered from heart failure with an impaired left ventricular systolic ejection fraction < 55%.

When comparing patients with and without heart failure, patients with heart failure are significantly older (77 (70-84) vs. 73 (63-83) years; p=0.011) and cardiovascular risk factors such as atrial fibrillation (50 (61.0%) vs. 81 (35.5%); p=< 0.001), peripheral artery disease (9 (11.0%) vs. 5 (2.2%); p=0.003), and coronary heart disease (44 (53.7%) vs. 41 (18.0%); p=<0.001) were significantly more frequent as well as chronic renal failure (27 (35.8%) vs. 35 (18.2%); p=0.003) (Table 1). Interestingly there were no differences in rates of pretreatment antiplatelet medication (21 (34.4%) vs. 53 (33.1%); p = 0.874) or oral anticoagulants (6 (17.1%) vs. 29 (18.1%); p = 0.153) in patients with heart failure compared to those without.

As expected, patients with heart failure had more cardiac comorbidities. Using echocardiography, we found that patients with heart failure suffer more often from heart valve insufficiencies (aortic insufficiency (39 (61.9%) vs. 83 (42.1%); p=0.009); mitral valve insufficiency (78 (95.1%) vs. 181 (81.2%); p=0.003); tricuspid valve insufficiency (69 (90.8%) vs. 164 (74.5%); p= 0.003)). When looking at the severity of valve insufficiencies especially, the difference becomes manifest. Patients with heart failure significantly suffer more often from severe mitral valve insufficiency (9 (90 %) vs. 1 (10 %); p < 0.001) and severe tricuspid valve insufficiency (8 (72.7%) vs. 3 (27.3%); p < 0.001).

Furthermore, dilatation of ventricles and atria were significantly more present in patients with heart failure comparing to those without (end diastolic left ventricular dilatation (15 (21.7%) vs. 2 (1%); p<0.001); left atrial dilatation (64 (91.4%) vs. 103 (58.5%); p<0.001); right ventricular dilatation (14 (40%) vs. 12 (10.5%); p<0.001); right atrial dilatation (7 (77.8%) vs. 8 (19%); p= 0.001)) (Table 2).

The neuroradiological characteristics did not differ between the two groups. There was no difference especially at the initial CCT-ASPECTS (8 (7-9) vs. 9 (8-10); p=0.155) or the collateral-status of the patients assessed by the Menon score (7 (5.5-8.5) vs. 7 (5-9); p=0.906).

Looking closer at the neurological characteristics of the severity of the stroke, there is no difference of the NIHSS at admission (15 (10.5-19.5) vs. 15 (10.5-19.5); p=0.085) and there is no difference regarding the NIHSS at discharge (6.5 (0-13) vs. 5 (0-10.5); p=0.324) or in the mRS at discharge (3 (1-5) vs. 3 (1-5); p=0.238) or at 90 days after stroke (3 (0-6) vs. 3 (1-5); p=0.380). After dichotomizing the mRS in patients with a favorable outcome as mRS of 0-2 and an unfavorable outcome for mRS 3-6 there is still no difference in patients with heart failure and without (32 (43.8%) vs. 105 (48.2%); p=0.521). We did not find a higher mortality either (9 (11.0%) vs. 17 (7.4%); p=0.313) (Table 3).

This is also reflected in the multivariate regression analysis where no influence of heart failure on outcome of the patients could be demonstrated (RR - 0.001; 95% CI: (-0.011-0.009); p=0.860) (Table 4).

## 4. Discussion

We could show that mechanical thrombectomy is as effective in patients with heart failure as in those without. Heart failure does not have an impact on neurological outcome after large vessel occlusion when treated with mechanical thrombectomy. These findings are coherent to the analysis of the VISTA cohort, in which it was shown that systemic thrombolysis is effective, regardless of the presence of heart failure [12]. Even though it had been shown that patients with heart failure experience a higher mortality and morbidity after stroke [14]. One possible explanation might be the impaired cerebral perfusion due to the compromised ejection fraction, leading to poorer baseline collateral status, which is associated with worse outcome in patients with LVO [11]. In our study, we could not find an impact of heart failure on the cerebral collateral status of the patients; patients with heart failure did show similar collateral status, measured by the Menon score, compared to patients without heart failure. Another factor often blamed for possible worse outcome of HF patients after stroke is that symptomatic intracranial bleedings after systemic thrombolysis are more common in patients with heart failure [15]. In contrast, we did not detect a higher rate of symptomatic intracranial bleedings after mechanical thrombectomy and the proportion of patients also receiving systemic thrombolysis was not different in either group. One statistical drawback might be that the rate of intracranial bleedings after mechanical thrombectomy was very low and potential differences may not have been detected due to the small number of intracranial bleedings.

The strength of this study is the sample size and the well characterized patient cohort with comprehensive data especially regarding the neurological and neuroradiological parameters, as well as a 90-day followup examination. A limitation is the retrospective character of the study. Furthermore, we only included patients with a reduced ejection fraction, and we did not include patients with heart failure with a preserved ejection fraction and diastolic dysfunction. Thus, there is a probability of inclusion of some of those patients into the control group, which might influence our results.

## 5. Conclusion

In our study heart failure is not associated with a poorer outcome or higher mortality after mechanical thrombectomy in LVO. The NIHSS at discharge and mRS at 90 days as well as the mortality are similar in the two groups of patients. Even though patients with heart failure suffer from a high morbidity and mortality in stroke, they seem to benefit from cerebral artery recanalization therapy just as patients without heart failure.

## Figures and Tables

**Table 1:** Baseline characteristics of patients with and without heart failure.

Baseline characteristics and cardiovascular risk factors	Normal systolic LV-ejection fraction	Heart failure with reduced ejection fraction	p-value
Age	73 (63-83)	77 (70-84)	0.011
Female	126 (53.8%)	41 (48.8%)	0.428
Male	108 (46.2%)	43 (51.2 %)	
Arterial Hypertension	182 (79.1%)	71 (85.5%)	0.203
Diabetes mellitus	53 (23.1%)	27 (32.9%)	0.082
Atrial fibrillation	81 (35.5%)	50 (61.0%)	< 0.001
Obesity	71 (33.0%)	33 (41.8%)	0.164
Dyslipidemia	101 (43.9%)	41 (50.6%)	0.298
Smoking	46 (22.7%)	12 (16.9%)	0.307
Peripheral artery disease	5 (2.2%)	9 (11.0%)	0.003
Coronary heart disease	41 (18.0%)	44 (53.7%)	<0.001
Chronic renal failure	35 (18.2%)	24 (35.8%)	0.003

**Table 2:** Echocardiological characteristics of patients with and without hear failure.

Echocardiographic characteristics	Normal systolic LV-ejection fraction	Heart failure with reduced ejection fraction	p-value
Posterior wall thickness (mm, median, IQR)	12 (10.5-13.5)	12 (10.5-13.5)	0.559
Left ventricular enddiastolic volume (ml, median, IQR)	43 (39.5-46.5)	46 (40-52)	0.001
Left ventricular endsystolic volume (ml, median, IQR)	25 (21-29)	31 (26-36)	0.001
Left atrial diameter (mm, median, IQR)	34 (29.5-38.5)	42 (37.5-46.5)	<0.001
Aortic sinus (mm, median, IQR)	30 (26.5-33.5)	30 (26.5-33.5)	0.454
Left ventricular ejection fraction (%,median, IQR)	55 (55-55)	44.5 (35.5-53.5)	<0.001
Left ventricular hypertrophy	138 (70.4%)	49 (69.0%)	0.826
Left ventricular dilatation	2 (1%)	15 (21.7%)	<0.001
Left atrial dilatation	103 (58.5%)	64 (91.4%)	<0.001
Right ventricular dilatation	12 (10.5%	14 (40%)	<0.001
Right atrial dilatation	8 (19%)	7 (77.8%)	0.001
Wall motion abnormalities	5 (2.6%)	53 (72.6%)	<0.001
Congested inferior vena cava	15 (8.8%)	22 (34.4%)	<0.001
Aortic insufficiency	83 (42.1%)	39 (61.9%)	0.009
Aortic valve stenosis	25 (15.1%)	10 (16.4%)	0.805
Mitral valve sclerosis	30 (26.5%)	10 (38.5%)	0.237
Mitral valve insufficiency	181 (81.2%)	78 (95.1%)	0.003
Pulmonary valve insufficiency	47 (40.9%)	14 (58.3%)	0.174
Tricuspid valve insufficiency	164 (74.5%)	69 (90.8%)	0.003
Thrombus in the Left atrial appendage	4 (4.4%)	2 (8.3%)	0.440
Endocarditis	1 (2.8%)	1 (10%)	0.391
Patent foramen ovale	33 (18.9%)	5 (11.4%)	0.275

**Table 3:** Neurological characteristics of patients with and without heart failure.

Neurological and neuroradiological characteristics	Normal systolic LV-ejection fraction	Heart failure with reduced ejection fraction	p-value
*Neurological Characteristics*			
NIHSS at admission (median, IQR)	15 (10.5-19.5)	15 (10.5-19.5)	0.085
NIHSS at discharge (median, IQR)	5 (0-10.5)	6.5 (0-13)	0.324
mRS at admission (median, IQR)	5 (4-6)	5 (4-6)	0.174
mRS at discharge (median, IQR)	3 (1-5)	3 (1-5)	0.238
mRS after 90 days (median, IQR)	3 (1-5)	3 (1-5)	0.380
Favorable Outcome	105 (48.2%)	32 (43.8%)	0.521
Intravenous rt-PA	160 (68.7%)	51 (60.7%)	0.185
Hemicraniectomy	9 (4.7%)	7 (10.3%)	0.098
Symptomatic Intracranial Hemorrhage	2 (1%)	3 (4.4%)	0.080
Mortality	17 (7.4%)	9 (11%)	0.313
*Toast criteria*			<0.001
Macroangiopathy	23 (12.2%)	4 (5.9%)	
Cardio-embolic	82 (43.6%)	53 (77.9%)	
other	11 (5.9%)	1 (1.5%)	
unknown	44 (23.4%)	5 (7.4%)	
ESUS	28 (14.9%)	5 (7.4%)	

*Neuroradiological Characteristics*			
*TICI-Scale*			0.177
0	15 (6.6%)	5 (6.0%)	
1	8 (3.5%)	8 (9.6%)	
2a	31 (13.5%)	7 (8.4%)	
2b	117 (51.1%)	39 (47%)	
3	58 (25.3%)	24 (28.9%)	
CT-ASPECTS (median, IQR)	9 (8-10)	8 (7-9)	0.155
Collateral Status (median, IQR)	7 (5-9)	7 (5.5-8.5)	0.906
Symptom onset to recanalization (min, median, IQR)	220 (159-281)	246 (176-316)	0.341
*Occlusion site*			0.326
Proximal carotid artery	8 (3.4%)	2 (2.4%)	
Distal carotid artery	39 (16.8%)	17 (20.5%)	
M1	135 (58.2%)	41 (49.4%)	
M2	25 (10.8%)	14 (16.9%)	
Basilar artery	24 (10.3%)	7 (8.4%)	
Posterior cerebral artery	1 (0.4%)	1 (1.2%)	
Anterior cerebral artery	0 (0.0%)	1 (1.2%)	

IQR: interquartile range, rt-PA: recombinant tissue plasminogen activator, NIHSS: National Institute of Health Stroke Scale, mRS: modified Rankin Scale, ESUS: embolic stroke of unknown source, TICI: thrombolysis in cerebral infarction, and CT-ASPECTS: computer tomography-Alberta Stroke program early CT score.

**Table 4:** Regression analysis regarding predictors of a good outcome (mRS 0-2) in patients with mechanical thrombectomy in LVO.

Outcome	RR	95% Confidence	Interval	p-value
Age	0.10	0.004	0.017	0.004
Occlusion side	-0.037	-0.178	0.104	0.606
Intravenous rt-PA	0.135	-0.059	0.328	0.171
Coronary heart disease	0.146	-0.066	0.357	0.174
Chronic renal failure	-0.109	-0.345	0.126	0.359
NIHSS at admission	0.016	0.000	0.032	0.044
Symptom onset to recanalization	0.001	0.000	0.002	0.021
TICI Scale	-0.125	-0.229	-0.021	0.019
CT-ASPECTS	-0.015	-0.085	0.054	0.665
Collateral Score	-0.027	-0.072	0.017	0.222
HFrEF	-0.001	-0.011	0.009	0.860
Left ventricular hypertrophy	-0.020	-0.206	0.165	0.828

RR: relative risk, rt-PA: recombinant tissue plasminogen activator, NIHSS: National Institute of Health Stroke Scale, TICI: thrombolysis in cerebral infarction, CT-ASPECTS: computer tomography-Alberta Stroke program early CT score, HRrEF: heart failure with reduced ejection fraction, mRS: modified Rankin Scale, and LVO: large vessel occlusion.

## Data Availability

The data used to support the findings of this study are available from the corresponding author upon request.
